# Study of the Vertical Transmission of COVID-19 by Using the World Health Organisation Protocol in a Tertiary Care Hospital in Eastern India

**DOI:** 10.7759/cureus.51926

**Published:** 2024-01-09

**Authors:** Vinita Singh, Archana Barik, Minakshi Mishra, Kumar Diwakar, Anisha Choudhary, Neelam Mehta

**Affiliations:** 1 Obstetrics and Gynaecology, Tata Main Hospital, Jamshedpur, IND; 2 Obstetrics and Gynaecology/DNB, Tata Main Hospital, Jamshedpur, IND; 3 Obstetrics and Gynaecology, Manipal Tata Medical College/Manipal Academy of Higher Education, Manipal, IND; 4 Pathology, Tata Main Hospital, Jamshedpur, IND; 5 Pediatrics, Tata Main Hospital, Jamshedpur, IND; 6 Pediatrics, Manipal Tata Medical College/Manipal Academy of Higher Education, Manipal, IND; 7 Obstetrics and Gynecology, Tata Main Hospital, Jamshedpur, IND; 8 Biochemistry, Tata Main Hospital, Jamshedpur, IND

**Keywords:** world health organization, mother to child transmission, vertical transmission, pregnancy, sars-cov-2, covid-19

## Abstract

Background: The World Health Organisation (WHO) has established criteria to diagnose vertical transmission in severe acute respiratory syndrome coronavirus 2 (SARS-CoV-2). This study aimed to determine the incidence of vertical transmission of SARS-CoV-2 using WHO criteria in a tertiary care centre in eastern India.

Methods: A hospital-based prospective observational study was conducted from June 2021 to February 2022 on women admitted for delivery with a positive nasopharyngeal (NP) swab and a SARS-CoV-2 real-time reverse-transcriptase polymerase chain reaction (RT-PCR) test. Following the delivery, the amniotic fluid (AF) and swab from the placenta were tested for SARS-CoV-2 by the Truenat test. The umbilical cord and maternal blood were analyzed to detect immunoglobulin M (IgM) and immunoglobulin G (IgG). The nasopharyngeal swabs of the newborns were tested for SARS-CoV-2 by RT-PCR.

Results: Forty-eight SARS-CoV-2-positive asymptomatic women were included in the study. Twenty-eight (58.3%) were delivered via cesarean section. Preterm delivery occurred in 13 (27.1%) cases. In only one case, vertical transmission was confirmed as the neonate had a positive nasopharyngeal SARS-CoV-2 RT-PCR test and the cord blood was IgM positive (suggesting an immune response in the neonate). The placenta was positive in three cases, and amniotic fluid was positive in two. However, vertical transmission was deemed unlikely in these cases as there was no evidence of immune response or viral persistence according to the WHO criteria. There was one stillbirth, and it tested negative for SARS-CoV-2.

Conclusion: This study strengthens the evidence of vertical transmission in COVID-19-positive asymptomatic mothers. The data suggest a low transmission rate.

## Introduction

The coronavirus disease 2019 (COVID-19) has affected millions of people all over the world since the first outbreak in December 2019 in Wuhan province, China. It was declared a pandemic by the World Health Organisation (WHO) on March 11, 2020 [1]. India is one of the most severely affected countries, with total infected cases crossing four crores and deaths accounting for more than five lakhs [2].

The manifestation of the disease varies from a mild upper respiratory tract infection to life-threatening pneumonia and acute respiratory distress syndrome (ARDS). Some may remain completely asymptomatic as well [3]. Studies have proven that certain populations, including the elderly, people with comorbidities, and immunocompromised individuals, have a higher susceptibility to developing severe forms of COVID-19 infection [4].

Though pregnant and non-pregnant females have similar risk profiles for COVID-19 infection, infected pregnant females, owing to their altered physiology, are at elevated risk of severe disease [5]. Subsequently, there is a likelihood of infection in the child born from the infected mother. Severe acute respiratory syndrome coronavirus-2 (SARS-CoV-2), the virus causing COVID-19 infection, tends to alter the immune response at the feto-maternal interface, resulting in adverse pregnancy outcomes including preterm birth and stillbirth, maternal morbidity, and mortality [6]. The possibility of vertical transmission is one of the biggest concerns.

Vertical transmission is defined as the transmission of the infectious pathogen from the mother to the foetus during the antepartum and intrapartum periods, or to the neonate during the postpartum period via the placenta in utero, body fluid contact during childbirth, or through direct contact owing to breastfeeding after birth [7]. The clinical concern for vertical transmission of COVID-19 arises from the fact that the angiotensin-converting enzyme-2 (ACE-2) receptor is present in the placenta, which is used by the SARS-CoV-2 virus to enter any cell [8]. The receptor is also expressed in the uterus, ovary, and vagina.

Initial reports from China have documented immunoglobulin M (IgM) antibodies in neonates born to mothers who had positive results for COVID-19, raising concerns for in-utero transmission [9]. As IgM does not cross the placenta, the presence of IgM in foetal serum suggests an immune response to an in-utero infection. Despite the evidence, earlier studies failed to detect any viral presence in neonates from infected mothers [10,11]. However, later studies conducted across the globe suggested evidence of vertical transmission of the SARS-CoV-2 virus [12-14]. However, the criterion for establishing vertical transmission was variable among the studies, and it was unclear whether the neonate got the infection in utero from the mother or acquired it after birth.

In December 2020, the WHO established criteria for defining mother-to-child transmission (MTCT) or vertical transmission of SARS-CoV-2 [15]. The criteria were last updated in November 2022. It described the possible timings of transmission at different stages of pregnancy, such as in-utero, peripartum, and postnatal (breast milk). The mother-to-child transmission in all three stages is assessed by the following three criteria: (i) evidence of maternal SARS-CoV-2 infection (symptomatic or asymptomatic) demonstrated by the viral presence or detection of IgM antibody in the nasopharyngeal (NP) sample from 14 days before delivery until 2 days after birth; (ii) evidence of in-utero foetal exposure to SARS-CoV-2, defined by detection of virus in either the amniotic fluid (AF), the placenta or the neonate within the first 24 hours postpartum; (iii) evidence of viral persistence confirmed by viral detection in the blood or nasopharyngeal sample of the neonate after 24 hours of birth or immune response in infants, characterised by detection of IgM antibodies in neonatal blood at 24 hours to 7 days. The vertical transmission is further classified into three categories: confirmed, possible, and unlikely, based on the fulfilment of the above criteria.

To date, only a few studies have used the WHO protocol to validate the mother-to-child transmission of SARS-CoV-2 [16]. We also followed the WHO protocol in this study to establish vertical transmission of COVID-19-infected mothers in a tertiary care hospital in eastern India. We primarily focused on the in-utero transmission of the virus.

## Materials and methods

The study population included SARS-CoV-2-infected pregnant women in the third trimester admitted to the COVID wing of the labour room for delivery at Tata Main Hospital from June 2021 to February 2022. The study has been approved by the institutional ethics committee at Tata Main Hospital, Jamshedpur, India, bearing approval no. TMH/AC/IEC/JUN/062A/2021. Informed consent was obtained from all women participating in the study. The infection was detected by a SARS-CoV-2 real-time reverse-transcriptase polymerase chain reaction (RT-PCR) test of the nasopharyngeal sample of the mother obtained during admission. Obstetric management of COVID-positive patients was carried out, and after delivery, the following five samples were collected: an amniotic fluid sample, a swab from the foetal surface of the placenta, umbilical cord blood and maternal blood, and a neonatal nasopharyngeal swab for the SARS-CoV-2 RT-PCR test within 24 hours of delivery. The amniotic fluid and placental swab were examined for SARS-CoV-2 by the Truenat COVID-19 test. The umbilical cord blood and maternal blood were tested for IgM and IgG antibodies.

Maternal age, gravida, parity, and gestational age at delivery were recorded. The associated medical comorbidities were also noted. Symptoms and signs of COVID-19 infection in study participants were documented, if any. In all patients, a complete blood count, blood sugar, serum creatinine, and C-reactive protein were taken. The mode of delivery, pregnancy outcome, maternal complications, and neonatal complications were recorded. Maternal complications related to the COVID-19 infection, such as supplemental oxygen therapy, invasive mechanical ventilation, intensive care unit (ICU) admission, maternal sepsis, and maternal mortality, were documented. Neonatal outcomes included birth weight, APGAR score, neonatal intensive care unit (NICU) admission, and NICU length of stay. Neonatal complications included transient tachypnoea of the neonate (TTN), respiratory distress syndrome (RDS), invasive mechanical ventilation, sepsis, and neonatal demise.

The nasopharyngeal swabs were obtained as per Centres for Disease Control and Prevention (CDC) guidelines [17]. After delivery, a full-thickness placenta, a swab from the fetal side of the placental bed, and amniotic fluid were obtained. Ten millilitres of venous umbilical cord blood and maternal blood were collected to study the presence of specific SARS-CoV-2 IgG and IgM antibodies. The nasopharyngeal swab of the newborns was collected immediately after delivery, according to international guidelines. Neonates were cleaned by dedicated nurses and they were not allowed to have skin-to-skin contact with their mother before the nasopharyngeal swab was collected. After the collection of the swabs, they were allowed to room in with their mothers. According to the World Health Organisation, mothers with SARS-CoV-2 infections can breastfeed their babies using appropriate precautions.

Detection of SARS-CoV-2 by RT-PCR

For SARS-CoV-2 detection by real-time RT-PCR, the RdRp and the E viral gene were amplified along with the human RNase P gene, which served as an internal control. The RT-PCR reactions were run on a true lab real-time quantitative micro-PCR analyzer (Molbio Diagnostics Private Limited, India), and the amplification curve was displayed on the analyzer screen. The number of amplification cycles required for the fluorescent signal to cross the threshold (i.e., exceed the background signal) is termed the cycle threshold (Ct). The Ct value is inversely proportional to the amount of target nucleic acid in the sample (viral load). In the case of negative samples, the amplification does not occur, and a horizontal amplification curve of the E gene and the RdRp gene is displayed on the screen during the test run. At the end of the test run, the E/RdRp gene “detected” or “not detected” result is displayed. In positive cases, a semi-quantitative (Ct value) result is also displayed on the screen. Ct values of less than 24 are considered to have a high viral load.

Detection of SARS-CoV-2 by Truenat

The Truenat assay is a chip-based, rapid molecular diagnostic test for detecting SARS-CoV-2. It works on the principle of real-time reverse transcriptase polymerase chain reaction based on Taqman chemistry. The RNA from the patient sample is first extracted using a TruePrep AUTO Universal cartridge-based sample prep device and kit (Molbio Diagnostics Private Limited, India). The extracted RNA, along with real-time PCR reagents, is added to a disposable microchip. The chip is then placed on the chip tray of the Truenat real-time micro-PCR analyzer for assay (Molbio Diagnostics Private Limited, Verna, Goa, India).

Detection of SARS-CoV-2 IgG and IgM antibodies

The presence of specific SARS-CoV-2 IgG and IgM antibodies was determined in maternal and umbilical cord serum by chemiluminescent immunoassay using the access SARS-CoV-2 IgG kit and access SARS-CoV-2 IgM kit in the Unicel DXI 800 Access Immunoassay system, Beckman Coulter. The SARS-CoV-2 IgM antibody titers were recorded as S/CO (signal/cutoff). S/CO < 1.00 was considered negative and S/CO ≥ 1.00 was considered positive. For SARS-CoV-2 IgG antibody titers, S/CO < 10.00 was considered negative and S/CO ≥ 10.00 was considered positive.

Interpretation of laboratory data in detecting in-utero mother-to-child transmission

We followed the WHO SARS-CoV-2 and pregnancy prospective cohort study generic protocol to determine the possibility of in-utero mother-to-child transmission. The three criteria we followed are as follows: (1) maternal infection detected by a positive SARS-CoV-2 RT-PCR test of the nasopharyngeal sample; (2) in-utero viral exposure detected by a positive RT-PCR nasopharyngeal sample of the neonate or viral detection in the AF and placenta by the Truenat COVID-19 test; (3) evidence of an immune response in the neonate as confirmed by the detection of an IgM antibody in umbilical cord blood. We did not check for viral persistence in neonates after 24 hours, as recommended by WHO criteria. To evaluate immune response, we did a serological examination to detect SARS-CoV-2-specific blood IgM and IgG in positive mothers. We also checked for IgG in cord blood in addition to IgM antibodies to study the passive transfer of immunoglobulins to the neonate. Based on the WHO criteria, in-utero vertical transmission is categorised as confirmed, possible, or unlikely. In utero transmission (IUT) is confirmed only when all three criteria are fulfilled.

Statistical analysis

All the data were collected in MS Excel (Microsoft® Corp., Redmond, WA). The continuous variables were expressed as mean (standard deviation) and ranges. Analysis of continuous variables was performed using parametric (Student’s t-test) or non-parametric (Mann-Whitney U-test) tests, depending on their distribution. The categorical variables were expressed as numbers and percentages. The analysis of categorical variables was done using the Chi-square test or Fisher’s exact test. All tests were one-sided, and a p-value < 0.05 was taken as statistically significant. The statistical analysis was done using SPSS software (Chicago, IL, USA), version 21.0.

## Results

In our study, 48 women tested positive for SARS-CoV-2 on admission from June 2021 to February 2022. The demographic parameters of the women are shown in Table 1. All the women were asymptomatic. Thirty-one patients had additional maternal comorbidities. There was one case of intrauterine foetal demise (IUFD), or stillbirth. Twenty-eight (58.3%) patients underwent caesarean section delivery. Table 2 shows various indications for caesarean sections. We did not observe any peripartum complications in any of the study participants. Preterm delivery occurred in 13 (27.1%) cases.

**Table 1:** Maternal variables. GDM: gestational diabetes mellitus; PIH: pregnancy-induced hypertension.

Variables		(n = 48)
Age (mean± SD)		29.29 ± 4.73
Parity	Primigravida, n (%)	23 (47.9)
	Multigravida, n (%)	25 (52.1)
Gestational age at delivery	Preterm, n (%)	13 (27.1)
	Term, n (%)	35 (72.9)
Mode of delivery	Cesarean delivery, n (%)	28 (58.3)
	Vaginal delivery, n (%)	20 (41.7)
Maternal morbidity	GDM, n (%)	6 (12.5)
	PIH, n (%)	7 (14.6)
	Obstetric cholestasis, n (%)	6 (12.5)
	Anaemia, n (%)	3 (6.3)
	Thrombocytopenia, n (%)	7 (14.6)
	Jaundice, n (%)	1 (2.1)
	Hepatitis, n (%)	1 (2.1)
	Stillbirth, n (%)	1 (2.1)

**Table 2:** Indications for caesarean section.

Indication	N = 28
Previous caesarean section, n (%)	10 (35.7)
Failed induction, n (%)	6 (12.5)
Foetal distress, n (%)	5 (10.4)
Oligohydramnios, n (%)	4 (8.3)
Placenta previa, n (%)	1 (2.1)
Foetal growth restriction, n (%)	1 (2.1)
Severe pre-eclampsia, n (%)	1 (2.1)

Table 3 shows neonatal outcome variables and complications. The average APGAR scores (appearance, pulse, grimace, activity, and respiration) at one minute were 8:8 ± 0.7, and at five minutes they were 8:9 ± 0.2. The APGAR score was less than seven in one neonate at five minutes of birth. Two preterm neonates developed RDS, and three-term neonates developed TTN. However, all these neonates with RDS or TTN tested negative for SARS-CoV-2. Low birth weight (weight < 2500 g) was identified in 14 neonates (29.16%). There was no neonatal mortality.

**Table 3:** Neonatal outcome variables. APGAR: appearance, pulse, grimace, activity, and respiration; SD: standard deviation; TTN: transient tachypnoea of newborn; RDS: respiratory distress syndrome.

Variables		n = 48
Birth weight (mean ± SD)		2.66 ± 0.47
Gender	Male, n (%)	26 (54.2)
	Female, n (%)	22 (45.8)
APGAR score	At 1 minute (mean± SD)	8:8 ± 0.7
	At 5 minutes (mean± SD)	8:9 ± 0.2
Neonatal complication	TTN, n (%)	3 (6.3)
	RDS, n (%)	2 (4.2)
	Sepsis	0
	Neonatal death	0

Table 4 shows the results of various SARS-CoV-2 tests done during the peripartum period. The placental foetal side swab, amniotic fluid, and nasopharyngeal swab from neonates were sent for a SARS-CoV-2 RT-PCR test to look for evidence of early, presumably in-utero infant viral exposure. The swab from the foetal side of the placenta was positive in three cases, out of which two had a vaginal mode of delivery and one had a caesarean section. The amniotic fluid swab was positive in two cases delivered through a caesarean section. The nasopharyngeal swab was positive in one neonate; however, neither the placental surface nor the amniotic fluid showed positivity in that neonate. IgM was studied in cord blood to look for evidence of viral persistence or immune response in infants. The value of IgM was >1 (positive) in three neonates, out of which one had a nasopharyngeal swab positive for SARS-CoV-2 RT-PCR. Therefore, in only one case (2%), vertical transmission was confirmed. Two neonates that tested IgM positive did not show any evidence of in-utero viral exposure (placenta, amniotic fluid, and nasopharyngeal sample were negative). We did not repeat the IgM test on these neonates after seven days of delivery. So, according to WHO criteria, intrauterine transmission was unlikely. Similarly, in the cases with positive amniotic fluid and placental swabs, the intrauterine transmission was unlikely as they did not show any evidence of viral persistence or immune response (IgM negative), and all neonates were asymptomatic. We did not find evidence of in-utero viral exposure or immune response in the stillbirth case. Immunoglobulin G (IgG) was detected in 20 (41.6%) COVID-positive pregnant women. In ten newborns, IgG was detected in cord blood, suggesting the passive transfer of immunity from mother to fetus. Three (out of the ten cases) neonates had passive immunity without their mothers having IgG positive, and one of them had placental swab and amniotic fluid positivity.

**Table 4:** Peripartum SARS-CoV-2 test results. RT-PCR: real-time reverse-transcriptase polymerase chain reaction.

SARS-CoV-2 test	n = 48
Maternal IgG positive n (%)	20 (41.7)
Neonatal IgG positive n (%)	10 (20.8)
Neonatal IgM positive n (%)	3 (6.3)
Neonatal nasopharyngeal RT-PCR positive n (%)	1 (2.1)
Placental surface Truenat positive n (%)	3 (6.3)
Amniotic fluid Truenat positive n (%)	2 (4.2)

## Discussion

Mother-to-child transmission, or vertical transmission of COVID-19, is a matter of debate and despite multiple studies addressing this issue, the mode and rate of transmission from the mother to the foetus are still controversial. The WHO has proposed a strict laboratory and clinical protocol for confirming vertical transmission of COVID-19. In this study, we have adhered to this protocol to establish the risk of mother-to-child transmission of SARS-CoV-2 in a tertiary care hospital in Eastern India.

**Figure 1:**
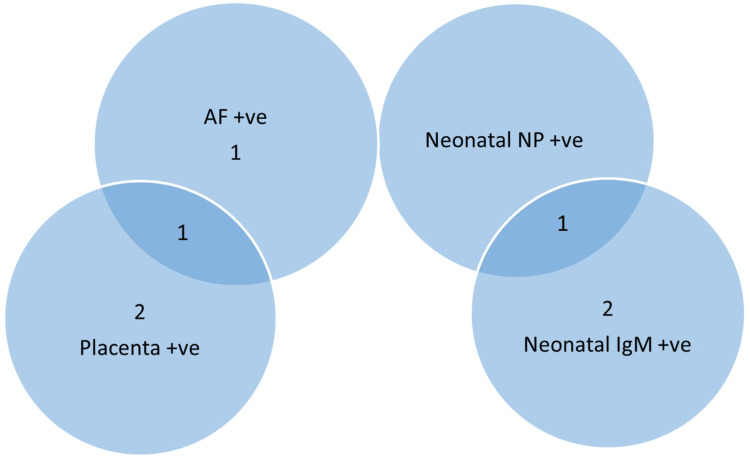
Venn diagram showing peripartum SARS-CoV-2 test results in SARS-CoV-2-positive women. In one neonate, the nasopharyngeal sample (in-utero viral exposure) and IgM were also positive (immune response in the neonate), confirming intrauterine transmission. Two neonates had an immune response (IgM positive) without evidence of in-utero viral exposure, suggesting intrauterine transmission was unlikely (repeat IgM was not done after one week). Placenta surface was exclusively positive in two cases, AF was exclusively positive in one case, and in the other case, both placenta and AF were positive, indicating in-utero infant viral exposure. However, none of these cases had a neonatal immune response, so intrauterine transmission was unlikely. AF: amniotic fluid; NP: nasopharyngeal sample; +ve: positive.

This study included 48 asymptomatic COVID-19-positive pregnant women in their third trimester of pregnancy admitted for delivery. The diagnosis of COVID-19 infection was done by an RT-PCR test of a nasopharyngeal sample. All the newborns were tested for SARS-CoV-2 by RT-PCR of a nasopharyngeal sample within 24 hours of delivery. The AF and the swab from the foetal side of the placenta were tested for viral presence by the Truenat COVID-19 test. The umbilical cord blood and the maternal blood were tested for SARS-CoV-2-specific IgG and IgM antibodies after delivery. Out of the 48 cases, only one (2%) showed strong evidence of intrauterine transmission as per WHO criteria. In four new-borns (8.3%), there was evidence of in-utero viral exposure (placental swab or amniotic fluid or both positive), but intrauterine transmission was unlikely in them as there was no evidence of immune response (umbilical cord blood IgM test negative) (Figure 1). The passive transfer of immunity was seen in 22% (n=45) of cases (IgG-positive). There was one stillbirth, which was unrelated to SARS-CoV-2 as there was no evidence of in-utero viral exposure.

Our study demonstrated that the risk of vertical transmission is low, especially for asymptomatic COVID-19-positive mothers diagnosed during the third trimester of pregnancy. Previous studies also suggested low rates of vertical transmission of COVID-19 in the third trimester of pregnancy [14,18,19]. The SARS-CoV-2 virus detection in amniotic fluid and placenta does not confirm infection of the newborn unless the neonate is symptomatic or there is evidence of an immune response. The timelines of maternal SARS-Cov-2 infection, the virus crossing the placenta into the amniotic fluid, and getting transmitted to the foetus are still grey areas [20]. Some studies have reported isolation of the virus in the placenta of women infected 4-17 days before delivery. The placenta is known to provide an immunological barrier that prevents the transmission of infection to the foetus [21], so the foetus may not get infected even if the virus is isolated from the placenta. In our study, it was difficult to know the exact timing of maternal infection as all were asymptomatic and tested for the virus 24 to 48 hours before delivery.

In some studies, rectal swabs have been used along with nasopharyngeal swabs to improve the detection rate of SARS-CoV-2 [16]. It is based on the suggestion that there is gastrointestinal shedding of the virus in paediatric patients [22]. In this study, we have taken only the nasopharyngeal swab.

**Figure 2:**
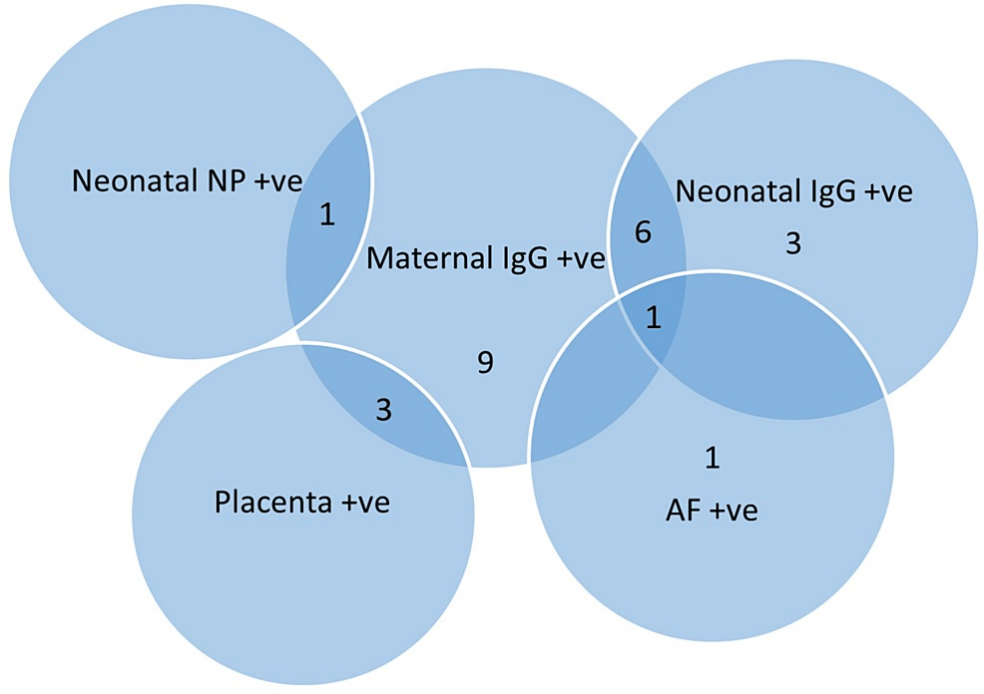
Venn diagram showing peripartum SARS-CoV-2 test results and women with positive immunoglobulin G status (n=20). Seven neonates had passive immunity out of 20 IgG-positive mothers, and one of them had positive amniotic fluid and placenta (not shown in the diagram), denoting possible in-utero transmission. Five neonates had a likelihood of in-utero exposure in mothers with positive maternal IgG (one had NP positive, three had placenta positive, and one had AF positive). NP: nasopharyngeal sample; AF: amniotic fluid; +ve: positive.

We examined the maternal IgG and cord blood IgG to look for a passive transfer of immunity from mother to fetus. The maternal IgG was positive in 41.7% (20 out of 48) of cases, out of which 35% of neonates had passive immunity (Figure 2). Surprisingly, in three cases, maternal blood was negative for IgG, but cord blood was positive. It probably suggested the persistence of acquired immunity in the foetus from an earlier SARS-CoV-2 infection in the mother.

A high viral load is usually associated with severe disease and probably increases the infectious rate [23]. Though all patients were asymptomatic in our study, we tried to investigate whether the viral load indicated by maternal nasopharyngeal RTPCR E gene Ct value had any impact on intrauterine transmission. Surprisingly, 35.4% of maternal NP samples had a high viral load (E gene Ct value < 24) despite being asymptomatic. We failed to detect any association between maternal viral load and intrauterine transmission or foetal immune response (Table 5). Sevilla-Montoya et al. had similar observations in their study on the vertical transmission of SARS-CoV-2 [16].

**Table 5:** Viral load and vertical transmission. NP: nasopharyngeal sample; AF: amniotic fluid.

Tests	High viral load (n=17)	Low viral load (n=31)	P-value
Neonatal NP positive n (%)	0 (0)	1 (3.22)	1
Placenta positive n (%)	0 (0)	3 (9.67)	0.5
AF positive n (%)	1 (5.88)	1 (3.22)	1
IgM positive n (%)	0 (0)	3 (9.67)	0.5

Previous studies have found an increased rate of preterm delivery in COVID-positive mothers [24,25]. In our study, all the mothers were asymptomatic, and the incidence of preterm delivery (27.1%) was not very high. A study with a larger sample size may give a clear picture regarding specific maternal complications of the SARS-CoV-2 infection. As per the WHO guidelines, the obstetric reasons indicated the mode of delivery. In our study, we did not find any impact of the mode of delivery on the rate of vertical transmission (Table 6).

**Table 6:** Mode of delivery and vertical transmission. NP: nasopharyngeal sample; AF: amniotic fluid.

Tests	LSCS (n=28)	VD (n=20)	P-value
Neonatal NP-positive n (%)	1 (3.57)	0	1
Placenta positive n (%)	1 (3.57)	2 (10)	0.56
AF positive n (%)	2 (7.14)	0	0.5
IgM positive n (%)	3 (10.71)	0	0.25

The strength of this study is that it strictly followed the WHO criteria for determining the vertical transmission of the SARS-CoV-2 infection. A few limitations of our study are that all the patients were asymptomatic, which might have impacted vertical transmission. Hence, there is a need for further research to determine the incidence in symptomatic mothers. The sample size in our study is small. The rectal swab was not tested for SARS-CoV-2 in neonates, which could have increased the detection rate. We did not check for evidence of viral persistence or immune response in the infant (repeat nasopharyngeal swab test or IgM status in the infant) after 24 hours of delivery to look for late infection and immune response as recommended by WHO.

## Conclusions

In conclusion, our study provides evidence to support the vertical transmission of COVID-19-infected asymptomatic mothers; however, the incidence was low. To confirm intrauterine transmission, there should be in-utero viral exposure (a positive nasopharyngeal sample of the neonate or viral detection in amniotic fluid or placenta) along with evidence of an immune response in the neonate (confirmed by the detection of an IgM antibody in umbilical cord blood). A mere viral presence in the placenta or amniotic fluid is not confirmatory of vertical transmission unless the neonate is symptomatic or there is evidence of an immune response in the neonate.

Currently, the evidence is limited regarding the precise timeline of vertical virologic transmission of COVID-19 infection in the third trimester of pregnancy. There are no specific pregnancy complications associated with maternal COVID-19 infection. A caesarean section may not be necessarily safer than vaginal delivery in preventing mother-to-child transmission of COVID-19. It is in line with the WHO recommendation on the mode of delivery, which suggests that delivery should be based on obstetric indications.
